# Size effects of graphene nanoplatelets on the properties of high-density polyethylene nanocomposites: morphological, thermal, electrical, and mechanical characterization

**DOI:** 10.3762/bjnano.11.14

**Published:** 2020-01-14

**Authors:** Tuba Evgin, Alpaslan Turgut, Georges Hamaoui, Zdenko Spitalsky, Nicolas Horny, Matej Micusik, Mihai Chirtoc, Mehmet Sarikanat, Maria Omastova

**Affiliations:** 1 Dokuz Eylul University, The Graduate School of Natural and Applied Sciences, Mechanical Engineering Department, Tinaztepe Campus, 35397, Buca, Izmir, Turkey; 2 Dokuz Eylul University, Engineering Faculty, Mechanical Engineering Department, Tinaztepe Campus, 35397, Buca, Izmir, Turkey; 3 GRESPI, Multiscale Thermophysics Lab, Université de Reims Champagne-Ardenne URCA, Reims, France; 4 Polymer Institute, SAS, Dúbravská cesta 9, 845 41 Bratislava, Slovak Republic; 5 Department of Mechanical Engineering, Ege University, 35100, Bornova, Izmir, Turkey

**Keywords:** electrical properties, graphene nanoplatelets, mechanical properties, polymer matrix composites (PMCs), thermal properties

## Abstract

High-density polyethylene (HDPE)-based nanocomposites incorporating three different types of graphene nanoplatelets (GnPs) were fabricated to investigate the size effects of GnPs in terms of both lateral size and thickness on the morphological, thermal, electrical, and mechanical properties. The results show that the inclusion of GnPs enhance the thermal, electrical, and mechanical properties of HDPE-based nanocomposites regardless of GnP size. Nevertheless, the most significant enhancement of the thermal and electrical conductivities and the lowest electrical percolation threshold were achieved with GnPs of a larger lateral size. This could have been attributed to the fact that the GnPs of larger lateral size exhibited a better dispersion in HDPE and formed conductive pathways easily observable in scanning electron microscope (SEM) images. Our results show that the lateral size of GnPs was a more regulating factor for the above-mentioned nanocomposite properties compared to their thickness. For a given lateral size, thinner GnPs showed significantly higher electrical conductivity and a lower percolation threshold than thicker ones. On the other hand, in terms of thermal conductivity, a remarkable amount of enhancement was observed only above a certain filler concentration. The results demonstrate that GnPs with smaller lateral size and larger thickness lead to lower enhancement of the samples’ mechanical properties due to poorer dispersion compared to the others. In addition, the size of the GnPs had no considerable effect on the melting and crystallization properties of the HDPE/GnP nanocomposites.

## Introduction

In recent years, electrically and thermally conductive polymer nanocomposites have attracted considerable attention because of their potential use in many industrial applications, such as aerospace, electronics, packaging, automotives, sensors, batteries, anti-statics, light-emitting devices, and corrosion-resistant coatings [1–2]. The addition of a conductive filler to the polymeric matrix is one of the easiest methods to produce these nanocomposites. Among conductive filler materials, carbon-based materials are attractive since they show significant enhancement of the properties of the nanocomposites at relatively low filler concentration compared to others [1,3–4]. Using graphene, a single-atom-thick structure of sp^2^ hybridized carbon atoms in a hexagonal arrangement, is of great interest due to its large specific surface area, 2D structure, and superior inherent properties, such as its thermal (1000–5000 W/mK [5]) and electrical conductivity (6000 S/cm [6]), and mechanical properties (a Young’s modulus of 1 TPa and a tensile strength of 130 GPa [7]). However, the mass production of graphene with high quality at a low cost is still challenging using available production technologies [8]. GnPs, formed by several graphene layers bonded together by van der Waals forces, are a potential alternative to graphene since they exhibit interesting properties and their fabrication cost is lower [8–9].

Numerous studies performed on nanocomposites with graphene or its derivatives have shown that the properties of nanocomposites depend on several factors, such as the polymer type, morphology, intrinsic properties, size and defect level of the filler material, production method, interface between filler and polymer, and others [10–12]. Although the size of GnPs is one of the most important factors, only few studies have investigated the size effect of GnPs on the properties of nanocomposites with various matrices, such as HDPE [13–15], polypropylene (PP) [8–9,16–17], epoxy [18–20], and polycarbonate (PC) [21] in the range of about 0.1–50 wt % (0.05–25 vol %). To the best of our knowledge, there has been no study on how the morphological, thermal, electrical, and mechanical properties of HDPE-based nanocomposites correlate with the size of GnPs in terms of thickness and lateral size. The objective of this study was to investigate the size effect of GnPs on the above-mentioned properties of HDPE-based nanocomposites. Considering the concentration of HDPE-based nanocomposites with GnP inclusions studied in the literature, the maximum concentration was up to 15 vol %. This study was carried out within the range of 0.92–13.92 vol % (2.05–26.81 wt %) of GnP concentration.

## Results and Discussion

### Characterization

**Figure 1:**
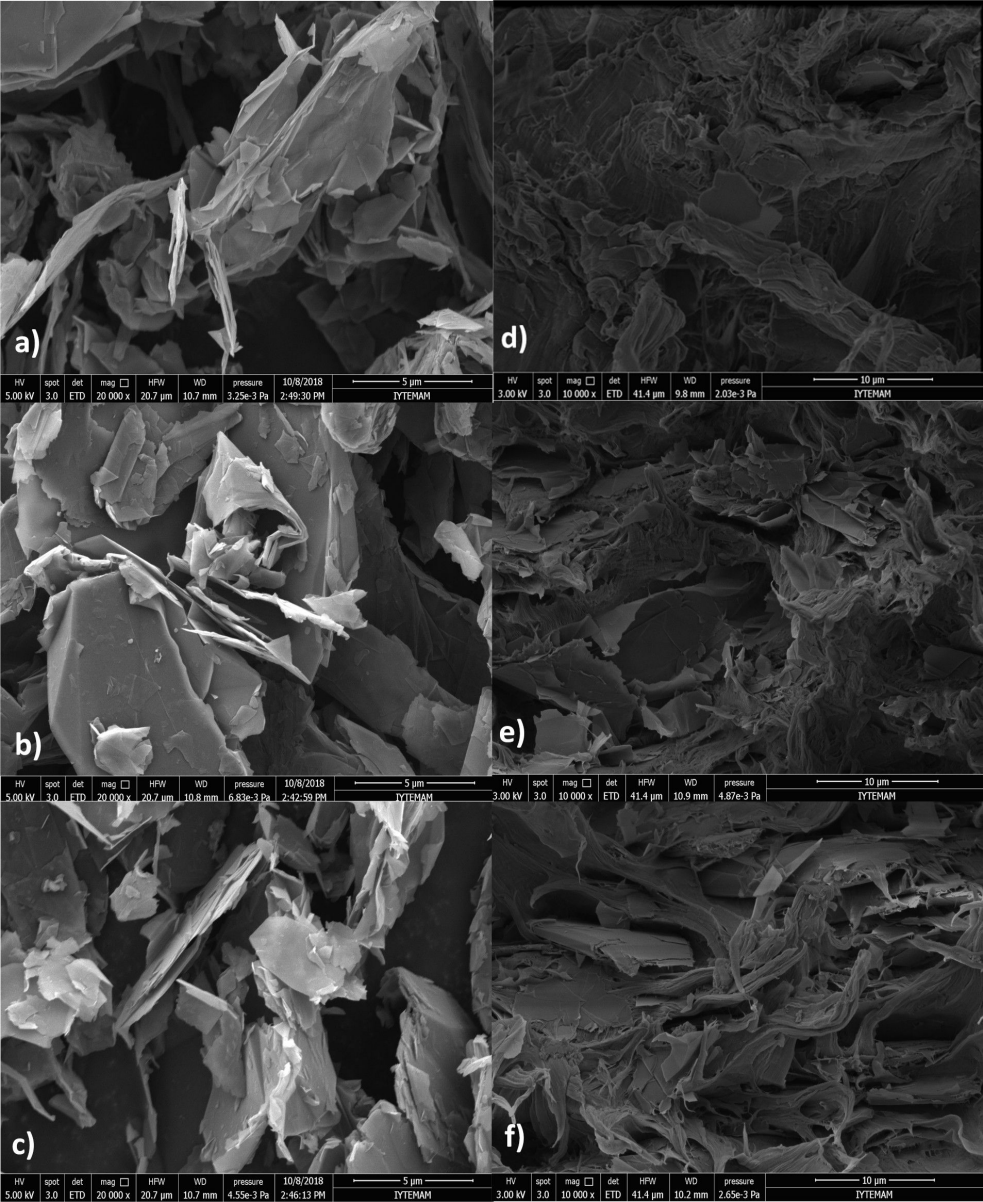
SEM images of a) G1, b) G2, c) G3, d) HDPE/G1 nanocomposite with 5.52 vol %, e) HDPE/G2 nanocomposite with 5.52 vol %, and f) HDPE/G3 nanocomposite with 5.52 vol %.

The SEM micrographs of each type of GnP and nanocomposites with 5.52 vol % GnPs are presented in Figure 1. The aggregated forms of the GnPs were observed due to their high surface energy. Figure 1 indicated that the particles of GnP types G1, G2, and G3 included many sharp edges and were irregularly shaped. As shown in Figure 1, it could be observed that the average lateral sizes of G1 and G2 were generally higher than those of G3, while G3 showed the lowest thickness. Furthermore, SEM images of the cryofractured surfaces of the HDPE/GnP nanocomposites with 5.52 vol % GnPs are shown in Figure 1d, Figure 1e, and Figure 1f to compare their structures. All types of GnPs were randomly and homogeneously distributed throughout the HDPE matrix where these layers separated the GnP nanoflakes. Two different dispersions of GnPs in HDPE were observed: separately dispersed and aggregated. The observed aggregates of GnPs, especially G3, may have been attributed to the large aspect ratio and high surface energy of the GnPs. In particullar, G1 nanoflakes were more isolated from each other, while G2 nanoflakes were found to be in more contact with each other. It is obvious from Figure 1 that G2 could form filler networks more easily than G1 and G3. From this study, it can be proposed that HDPE-based nanocomposites with G2 may show a greater number of GnP pathways and consequently higher electrical and thermal conductivity values. Additionally, G3 was seen to have better dispersion when compared to G1 containing the HDPE nanocomposites.

**Figure 2:**
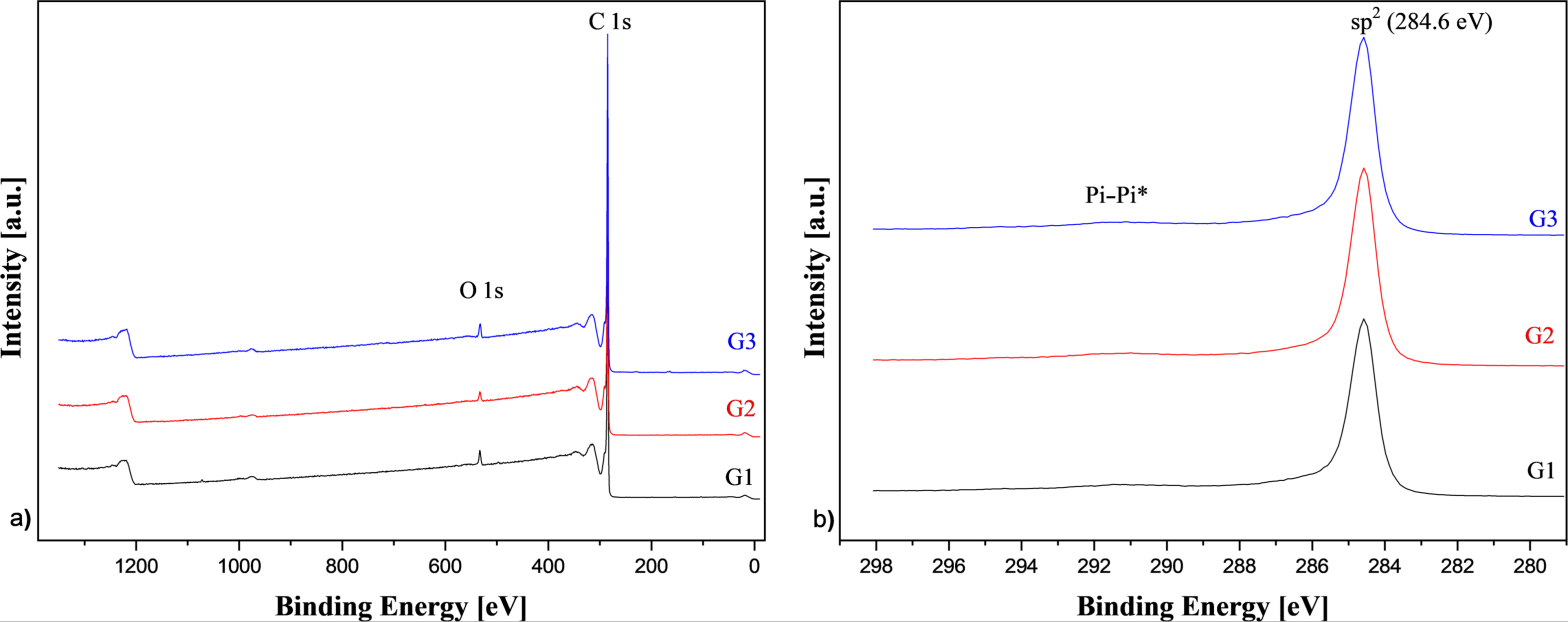
XPS: a) survey and b) C 1s high-resolution spectra for G1, G2, and G3.

XPS sample analysis gave more information on the GnPs’ chemical composition. The XPS results of GnPs are shown in Figure 2 and Table 1. The GnPs generally showed a strong signal for carbon (C 1s at ≈284.6 eV) and contained a small amount of oxygen (O 1s at ≈531–534 eV). The C 1s peak centered at ≈284.6 eV, with an asymmetry towards higher binding energies, which is characteristic for an sp^2^ carbon atom. The broad peak observed at ca. 291 eV (labeled ‘Pi–Pi*’ in Figure 2b) occurred due to the existence of delocalized π electrons (conduction electrons) available for shake-up events following core electron photoemission (Thermo Fisher Scientific Avantage Data System 5.9904; Thermo Fisher XPS: Knowledge Base). In Table 1, the deconvolution fit for the C 1s and O 1s signals is also shown. Besides graphitic carbon signals (sp^2^, centered at ca. 284.6 eV), signals for C–O (ca. 286.4 eV), C=O (287.4 eV), and OC=O (289.2 eV) groups were detected. These signals correspond to the O 1s signals of C=O aromatic (C=O_ar_, centered at ca. 531.2 eV), C=O aliphatic (C=O_al_, centered at ca. 532.2 eV), C–O (ca. 533.5 eV), and O_2_C=O (ca. 535.8 eV) groups. The C 1s signals of GnPs were approximately the same (Table 1 and Figure 2b). It was clear that G2 showed the highest atomic content of C 1s (98.4%), and the surface of G2 was the least oxidized one. This reduced degree of oxidization of G2 was easily indicated by the low amount of carbonyl (C=O_ar_) in the case of the O 1s signal. There was also a small amount of surface contamination present in the case of G1, with 0.1 atom % of Si 2p at ca. 103.6, corresponding to siloxanes, and 0.1 atom % sodium (Na 1s at ca. 1072.1 eV). For G3, some contamination with sulfur was observed (0.3 atom %, S 2p at ≈164–169 eV). However, the small differences in the GnPs’ surfaces’ chemical composition did not affect the properties of the HDPE-based nanocomposites.

**Table 1:** Apparent surface chemical composition of GnPs, as determined by XPS.

sample	surface chemical composition (atom %)		
	C 1s sp^2^/sp^3^/C–O/C=O/OC=O/π–π*	O 1s C=O_ar_/C=O_a_l/C–O/O_2_C=O	S 2p/Na 1s/Si 2p

G1	97.2 66.5/21.0/2.2/1.1/0.4/5.9	2.6 0.7/1.0/0.7/0.2	–/0.1/0.1
G2	98.4 67.3/21.6/1.9/1.0/0.4/6.2	1.6 0.3/0.8/0.4/0.1	–/–/–
G3	97.3 65.1/21.8/2.1/1.4/0.6/6.3	2.4 0.8/0.8/0.7/0.1	0.3/–/–

**Figure 3:**
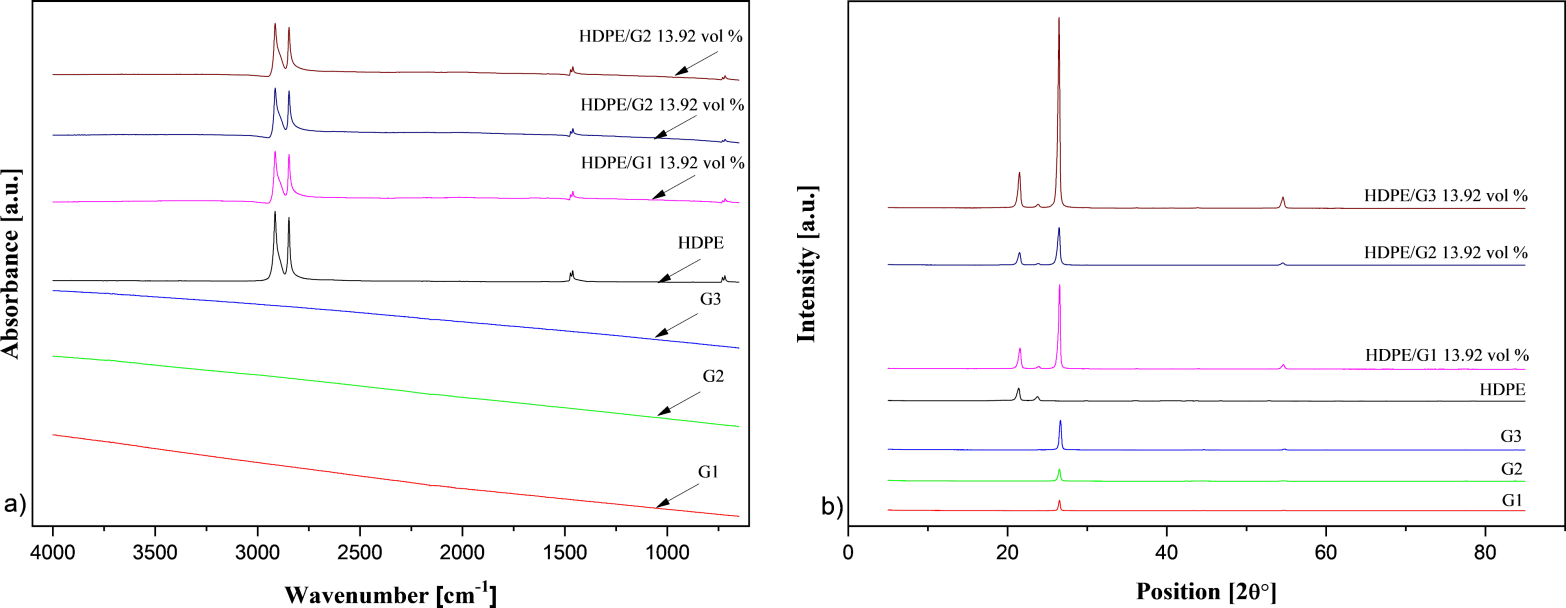
a) FTIR spectrum and b) XRD pattern of the GnPs, pure HDPE, and HDPE/13.94 vol % GnP nanocomposites.

The results of FTIR analysis are shown in Figure 3a (further details can be found in Figures S1–S7, Supporting Information File 1). The spectrum of HDPE showed two strong peaks at 2914.86 cm^−1^ and 2847.37 cm^−1^, respectively, and four weak peaks at 1472.59 cm^−1^, 1462.11 cm^−1^, 730.28 cm^−1^, and 718.84 cm^−1^. The peaks at ≈2914 cm^−1^ and ≈2847 cm^−1^ were assigned to alkyl (C–H) stretch vibrations. The peaks at the wave numbers ≈1471 cm^−1^ and ≈1462 cm^−1^ were related to the –CH_2_ group’s binding vibrations. The absorption peaks at ≈730 cm^−1^ and ≈718 cm^−1^ were attributed to aromatic C–H bending. As seen in Figure 3a, all GnPs did not display any peak assigned to functional groups on their surface. The curves of HDPE and HDPE/GnP nanocomposites had almost the same absorption peaks. The size and content of the GnPs caused no change in the characteristic peaks of the HDPE matrix.

Additionally, the crystalline structures of GnPs, HDPE, and HDPE/GnP nanocomposites were studied, and their XRD patterns are shown in Figure 3b (see also Figures S8–S10 in Supporting Information File 1). All types of GnPs showed a sharp peak at 2θ ≈ 26.5° and a weak peak at 2θ ≈ 54.6° corresponding to the (002) and (004) carbon reflections, respectively. The size of the GnPs did not cause a significant difference in the GnPs’ crystalline structure. The sharp peak located at 2θ ≈ 26.5° was characteristic for hexagonal GnPs, with a d-spacing of 0.336. Two strong peaks centered at 2θ = 21.38° and 2θ = 23.75°, corresponding to the (110) and (200) reflections of the HDPE orthorhombic phase, respectively, and two weak peaks centered at 2θ = 29.93° and 36.13°, corresponding to the (210) and (020) reflection planes, respectively, were clearly seen in the XRD pattern of pure HDPE. Similar HDPE peaks were observed by Sever and co-workers [22] and Wang and co-workers [23]. In the XRD patterns of the HDPE/GnP nanocomposites, the intensities of the peaks at 2θ ≈ 21.4° and 2θ ≈ 23.8° decreased with increasing GnP loading, while the intensities of the peaks at 2θ ≈ 26.5° and 2θ ≈ 54.6° increased. The XRD pattern of the HDPE-based nanocomposites exhibited a mix of the peaks appearing for HDPE and the GnPs. In the XRD patterns of the HDPE/GnP nanocomposites, the intensities of the peaks at 2θ ≈ 21.4° and 2θ ≈ 23.8° decreased with increasing GnP loading, while the intensities of the peaks at 2θ ≈ 26.5° and 2θ ≈ 54.6° increased. This was due to incorporation of GnP particles into the HDPE matrix. It was observed that all planes, which displayed considerable peak growth with the existence of GnPs, were in the general form of the (00x) type, being crystallographically in line with the GnP (002) plane. It has been indicated that GnPs may act as nucleation sites and that nucleation starts around the GnPs [8]. Figure 3b shows that the size of the GnPs had only a slight effect on the reflection peaks of the HDPE/GnP nanocomposites, but the peak’s intensity was influenced stronger.

### Electrical conductivity

**Figure 4:**
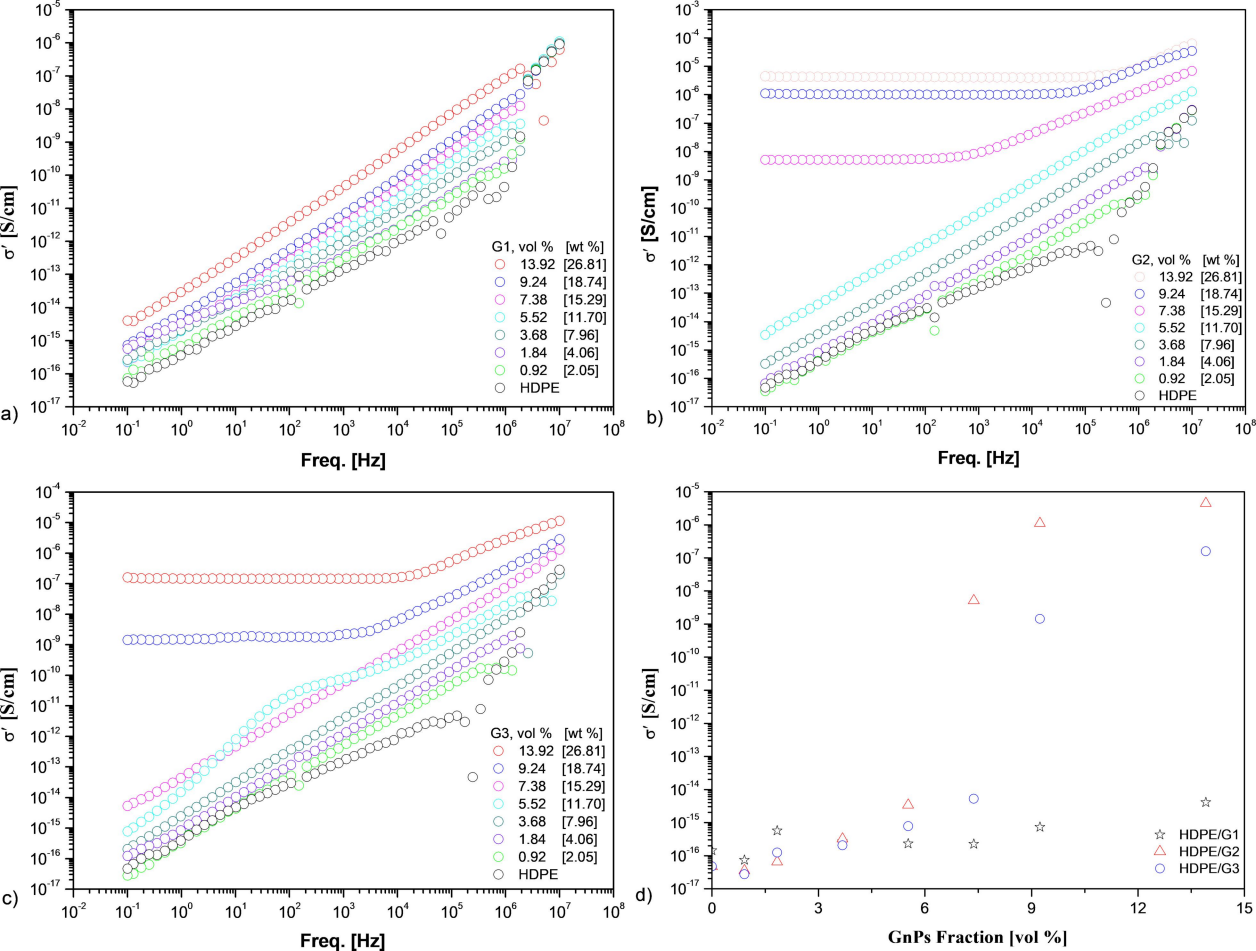
Real part of AC electrical conductivity vs frequency for a) HDPE/G1, b) HDPE/G2, and c) HDPE/G3 nanocomposites as well as d) σ´_AC_ at 0.1 Hz vs vol % of GnP for the HDPE/GnP nanocomposites.

Figure 4a–c demonstrate the AC conductivity (σ´_AC_) of all HDPE/GnP nanocomposites, measured at room temperature as a function of frequency. Pure HDPE is an insulating material that has a characteristic dielectric behavior corresponding to a linear increase in AC conductivity with increasing frequency [24]. The electrical conductivity of the nanocomposites increased with increasing amount of GnP. The reason for this increase may have been that a greater number of electrons was conducted through the HDPE/GnP interface because frequency facilitated the hopping of electrons [20]. For the HDPE/G1 nanocomposites, no significant enhancement of the electrical conductivity was observed. Figure 4a shows that the HDPE/G1 nanocomposites demonstrated the same linear behavior as pure HDPE for all GnP concentrations. A similar behavior was also exhibited by the HDPE/G2 and HDPE/G3 nanocomposites below a certain concentration, called the percolation threshold, which is defined as the minimum filler concentration where the insulating material is converted to a conductive or semiconductive material [25]. Above the percolation threshold, the dependence of the electrical conductivity on the frequency showed two different parts: i) the conductivity did not depend on the frequency up to a critical value, followed by ii) a regime in which the conductivity was dependent on the frequency. The first part was linked to the transfer of charge carriers by direct contacts between the GnP filler, while the second part reflected the conductivity due to hopping and tunneling of electrons between adjacent particles [26]. It was apparent that the critical frequency shifted towards higher frequencies, and the AC conductivity increases with increasing GnP loading.

Figure 4d shows the variation in σ´_AC_ at 0.1 Hz for pure HDPE and the HDPE/GnP nanocomposites as a function of GnP content. Pure HDPE demonstrated a characteristic insulator behavior, with an electrical conductivity of 4.68 × 10^−17^ S/cm, which was typical for polymers. For all GnP loadings, the HDPE/G1 nanocomposites were insulating, while it could clearly be seen that at GnP concentrations higher than 5.52 vol % for G2 and 7.38 vol % for G3, respectively, the HDPE-based nanocomposites became conductive. The nanocomposites showed a nonlinear increase in conductivity as a function of filler content. The HDPE/G2 and HDPE/G3 nanocomposites exhibited a characteristic S-shaped curve divided in three parts: insulating, transitioning, and conducting [27]. As seen in Figure 4d, the electrical conductivity of the nanocomposites was approximately the same up to the percolation threshold for each type of nanocomposite. As the GnP loading reached the percolation threshold, there was an abrupt increase in electrical conductivity because a conductive network was formed. The percolation threshold varied in terms of the size of GnPs, as mentioned by Bakir and co-workers [12]. The percolation thresholds of G2 and G3 were 5.52 vol % and 7.38 vol %, respectively. However, the percolation threshold was not observed for the HDPE/G1 nanocomposite, which should appear at higher concentrations. Nevertheless, with a filler concentration of 13.92 vol %, the electrical conductivity of the HDPE/G2 nanocomposite was approximately one order of magnitude higher than that of the HDPE/G3 nanocomposite with the same filler content.

Furthermore, one of the most important parameters for the percolation threshold was the aspect ratio of the GnPs [28]. However, the aspect ratio was not the only important parameter. As described by Horny and co-workers [29], for a given lateral size, an optimal thickness exists that gives the highest thermal conductivity. It could be assumed that this is also valid for electrical conductivity. Here, the optimum value for thickness seemed to be closer to 5–8 nm than 50–100 nm when comparing GnPs with the same lateral size (i.e., G1 and G3) because the lower percolation threshold was observed for G3 (Figure 4). It is worthwhile to mention that the efficiency of G2 in enhancing the electrical conductivity of HDPE was even better than that of G1 and G3. Finally, compared to GnPs with the same thickness but different lateral sizes, GnPs with larger lateral sizes exhibited a lower percolation threshold and higher enhancement of their electrical conductivity, as they formed a more easily conductive network and a better dispersion. Our results were in agreement with those of Jun and co-workers [8], who also found that the percolation threshold of the PP-based nanocomposites decreased with increasing lateral size of GnPs, while the thermal conductivity of samples increased. However, Al-Imran and co-workers. [9] and Jiang and Drzal [13] claimed that the percolation threshold values of the nanocomposites was almost the same regardless of GnP size. Additionally, compared in terms of thickness, the nanocomposites with thicker GnPs had a lower electrical conductivity due to a poorer dispersion level, confirming the hypothesis of the existence of an optimum thickness for a given lateral size.

### Thermal conductivity

**Figure 5:**
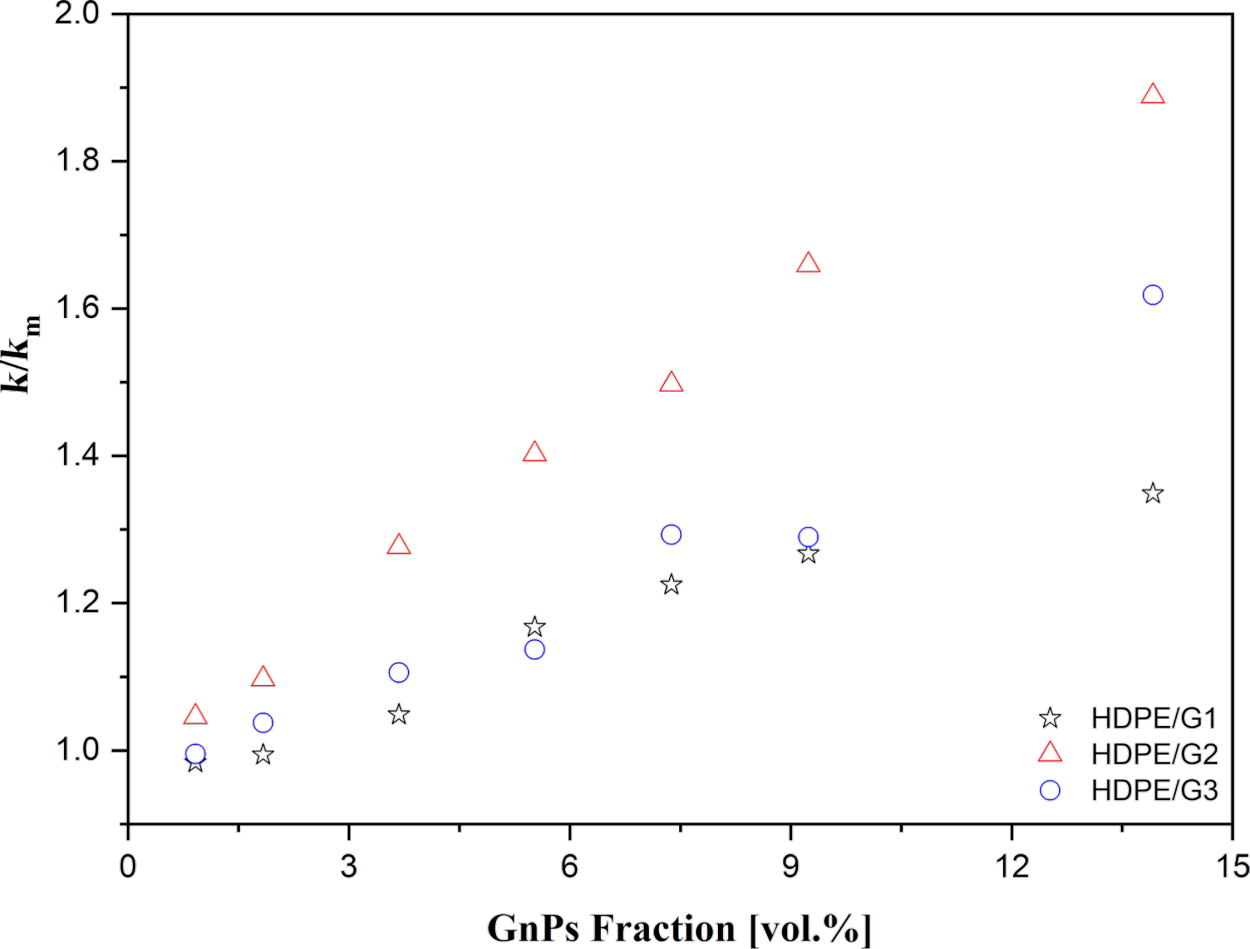
The relative thermal conductivity values of HDPE-based nanocomposites with G1, G2, and G3 fillers (*k*_m_ was equal to the thermal conductivity of the matrix).

Thermal diffusivity measurements were performed by the photothermal radiometry (PTR) method, and the thermal conductivity (*k* = α⋅ρ⋅*C*_p_) of nanocomposites was calculated by combining the experimentally measured thermal diffusivity (α) data, the theoretical specific heat (*C*_p_), and the density (ρ) values. These theoretical values were determined by mixing law theory. *C*_pm_ and *C*_pf_ were taken as 1832.9 J/(kg·K) [3] and 700 J/(kg·K) [30], respectively, and ρ_m_ and ρ_f_ were used as 964.9 kg/m^3^ [3] and 2186.7 kg/m^3^ [31–33], respectively. The subscripts m and f referred to the matrix and filler material, respectively. The results of the relative thermal conductivity measurements are given in Figure 5.

As a reference, the thermal conductivity of pure HDPE is 0.386 W/mK [3]. In Figure 5, regardless of the GnP size, the thermal conductivity of the nanocomposites increased linearly with the addition of GnPs due to the high thermal conductivity and large contact area of the GnPs. No thermal percolation threshold was observed for all types of nanocomposites. The thermal conductivity enhancement was stronger for the HDPE/G2-based nanocomposite than for those with G1 and G3 at any given GnP concentration. For example, with a GnP content of 13.92 vol %, the thermal conductivity of the HDPE/G2 nanocomposite was 0.729 W/mK, while for the HDPE/G1 and HDPE/G3 nanocomposites, it was enhanced up to 0.521 W/mK and 0.625 W/mK, respectively. Therefore, G2 demonstrated a better enhancement of 89% compared to pure HDPE, while G1 and G3 showed improvements of 35% and 62%, respectively. Moreover, despite the high intrinsic thermal conductivity of GnPs, the thermal conductivities of the HDPE/GnP nanocomposites increased moderately. The lower than expected thermal conductivity enhancement was mainly ascribed to the high interfacial thermal boundary resistance (TBR) between the HDPE matrix and the GnP fillers. TBR is a physical parameter causing lower thermal conductivity by hindering phonon transfer between the HDPE matrix and the GnPs fillers [21,34–35].

Furthermore, GnPs with larger lateral size showed a larger enhancement of the thermal conductivity. A similar lateral-size effect of GnPs was also observed in earlier studies [13,18,21]. One reason was that the fillers could more easily contact each other to create a more conductive network within the nanocomposites, resulting in a higher thermal conductivity [13,21,36]. Another reason was that a small lateral size increased phonon scattering at the matrix-bonded interface, resulting in a lower thermal conductivity of the nanocomposites [21]. Additionally, for the same lateral size, G3 showed a slight enhancement of the thermal conductivity compared to G1 (the enhancement became more effective at 13.92 vol % loading). Based on the obtained results, it is suggested that, for a given lateral size, the maximum thermal conductivity is obtained at the optimum thickness of GnPs. For a fixed lateral size (5 µm), the optimum thickness was approximately 5–8 nm over 50–100 nm. The reason may have been that thinner GnPs could couple well to the HDPE matrix and form a more effective thermal conductive network. Hovewer, Figure 5 displays that the thermal conductivity of HDPE/G3 nanocomposites slightly decreased up to 1.84 vol % GnPs loading. The explanation is that G3 tended to aggregate, resulting in a decreased contact area between HDPE and G3, as mentioned by Huang and co-workers [5].

Subsequently, the enhancement of the thermal conductivity of the HDPE-based nanocomposites could be increased by optimizing the lateral size and thickness of GnPs at a fixed GnP concentration. Based on these results, the lateral size of GnPs was a more important parameter than the thickness of GnPs in terms of thermal conductivity enhancement. The thermal conductivity of the nanocomposites with a larger lateral size could be further increased by forming a more effective thermal conductive pathway and reducing phonon scattering at the matrix/filler interface. In addition, to obtain the same thermal conductivity enhancement, larger amounts of thicker GnPs were required rather than thinner GnPs. Consequently, as previously mentioned, it was clear that the highest enhancement of the electrical and thermal properties was achieved by HDPE/G2 nanocomposites. The reason may have been that G2 could establish a more effective conducting network and cause an increased filler contact. In addition, G2, with higher lateral size, exhibited a better phonon diffusion mechanism along the conductive network.

### Mechanical properties

**Figure 6:**
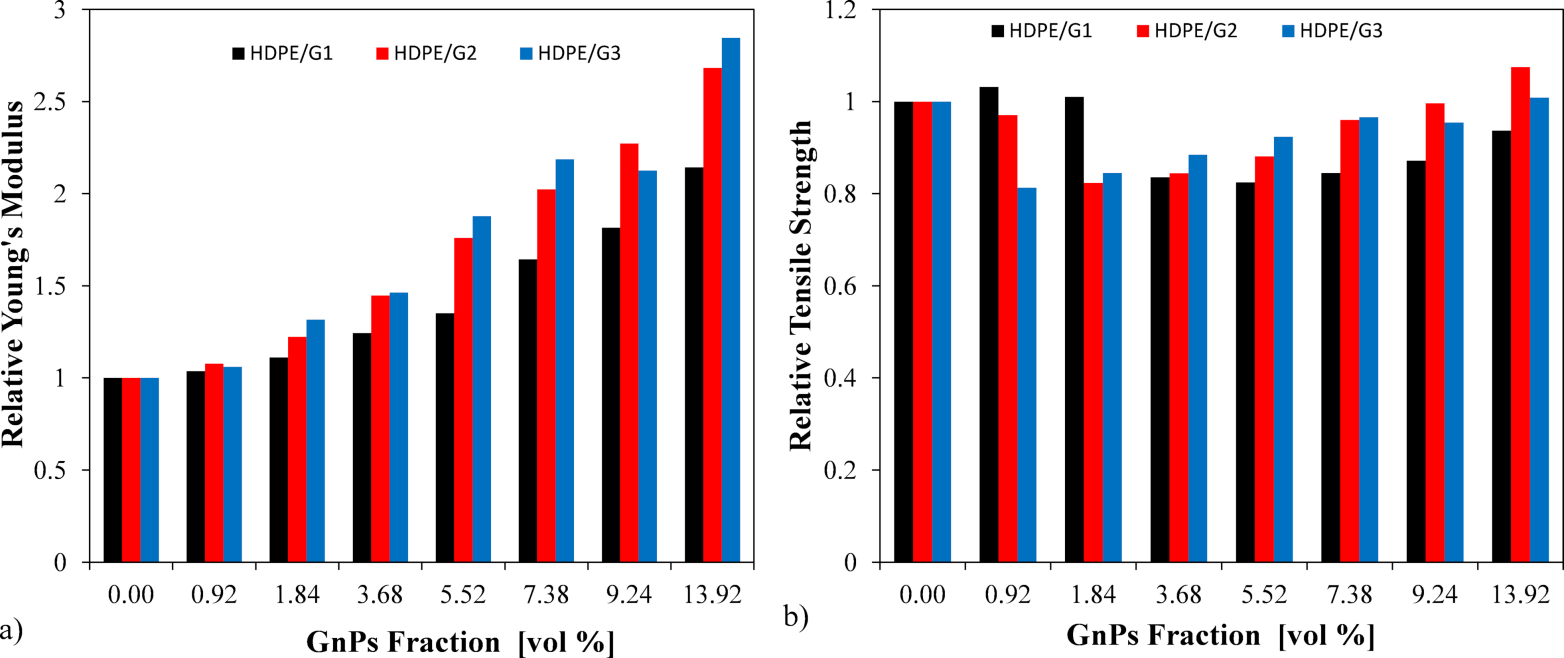
Relative mechanical properties of the HDPE/GnP nanocomposites: a) Young’s moduli and b) tensile strengths.

Following the electrical and thermal characterization, Figure 6 shows the relative Young’s moduli of the pure HDPE and HDPE/GnP nanocomposites. The Young’s moduli of the nanocomposites increased with increasing GnP loading, regardless of the GnP size. This may have been due to the extraordinary Young’s moduli of GnPs, their high aspect ratio, good dispersion, and good interfacial adhesion between GnPs and HDPE, which restricted the polymer chain mobility under the load [6,37]. The influence of GnP loadings increased the crosslink ratio and hindered the molecular motion of the matrix [6]. When the GnP loading was 13.92 vol %, the enhancements of the Young’s moduli of the HDPE/G1, HDPE/G2, and HDPE/G3 nanocomposites were 114.36%, 168.21%, and 184.58%, respectively. As seen in Figure 6a, the highest Young’s modulus was achieved by the HDPE/G3 nanocomposite, with an increment of up to 2.37 GPa. It can be seen that the Young’s modulus of the HDPE/G1 nanocomposite was the lowest, while the value of the Young’s modulus of the HDPE/G3 nanocomposite was the highest.

First, the tensile strength of the HDPE/G2 and HDPE/G3 nanocomposites decreases with increasing GnP loading in the range of 0–1.87 vol %, while the HDPE/G3 nanocomposites showed a decrement in tensile strength in the range of 1.87–5.52 vol % of GnPs. These decrements were 17.68% for the HDPE/G2 nanocomposite and 15.53% for the HDPE/G3 nanocomposite using 1.87 vol % GnP filler. The tensile strength depends on the filler content in a complex way [38]. The presence of filler can suppress the drawability of the polymer chains and increase the concentration of defects, which can then result in a decreased tensile strength. On the other hand, the filler can reinforce the polymer matrix, and this reinforcing effect becomes more obvious at higher concentrations (≥7 vol %). The nonlinear behavior of the tensile strength is then a consequence of these effects. With 37.27 MPa, the tensile strength of the HDPE/G2 nanocomposite seemed to be the highest among all HDPE/GnPs, and demonstrated a 7.47% enhancement compared to HDPE. On the other hand, the HDPE/G1 nanocomposite demonstrated a decrease of 6.33% in tensile strength, while the HDPE/G3 nanocomposite displayed an increase of 0.83% compared to pure HDPE.

It was found that G3, with a smaller size and thickness (larger aspect ratio and surface area), showed an effective enhancement of the Young’s moduli of the nanocomposites owing to the fact that a larger surface area provided a more efficient stress transfer between GnPs and the HDPE matrix, while G2, with a larger size and thickness, demonstrated an effective improvement of the tensile strength of the nanocomposites because G2 achieved a better dispersion with less agglomeration, as seen in SEM images. According to the obtained results, the dispersion of GnPs in the HDPE matrix was a more important parameter for the tensile stress than others; however, the surface area of GnPs was a more critical factor. In the literature, a similar type of observation was made by Liang et al. [16–17] and Jun and co-workers [8] where it was reported that GnPs with smaller lateral sizes were more favorable for enhancing the mechanical properties of PP-based nanocomposites.

### Thermal properties

The crystallization temperatures (*T*_c_), melting temperatures (*T*_m_), heats of fusion (Δ*H*_f_), and crystallinities (*X*_c_) of pure HDPE and the HDPE/GnP nanocomposites are given in Table 2. *T*_c_ for pure HDPE was found to be 116.30 °C, whereas for all HDPE/GnP nanocomposites, *T*_c_ increased by approximately 5 °C at 13.92 vol % GnP content due to the nucleation effect of the GnPs. It was clearly seen that *T*_m_ of all HDPE/GnP nanocomposites ranged from 130.43 °C to 132.17 °C, which almost equaled that of pure HDPE (130.09 °C). For all samples, *X*_c_ first increased and then decreased with increasing GnP content. This was due to a compromise between the nucleating and retarding effects of GnPs on the HDPE matrix, as mentioned by Jiang and Drzal [13]. It was clear that Δ*H*_f_ of all HDPE/GnP nanocomposites decreased by approximately 25% to a minimum value with 13.92 vol % GnP loading. The crystallinity degree of the pure HDPE was ≈70% and increased up to ≈71% with 13.92 vol % G1 and G2, respectively, and up to ≈72% with 13.92 vol % G3. This increase was relatively inconsiderable considering the GnP content added to the HDPE matrix. These results clearly demonstrate that while there was a slight effect of the GnP loading on *T*_m_ and *X*_c_, a significant effect on *T*_c_ and Δ*H*_f_ was observed. It could be observed that the size of GnPs induced little difference in these properties of the HDPE-based nanocomposites.

**Table 2:** DSC results for the HDPE/GnP nanocomposites.

GNP (vol %)	*T*_m_ (°C)			*T*_c_ (°C)			Δ*H*_f_ (J/g)			*X*_c_ (%)		
	HDPE/G1	HDPE/G2	HDPE/G3	HDPE/G1	HDPE/G2	HDPE/G3	HDPE/G1	HDPE/G2	HDPE/G3	HDPE/G1	HDPE/G2	HDPE/G3

0	130.09	130.09	130.09	116.30	116.30	116.30	205.80	205.80	205.80	70.24	70.24	70.24
1.84	131.82	132.17	132.01	117.66	117.31	117.33	200.60	205.20	204.20	71.36	73.00	72.64
5.52	130.89	131.63	132.05	118.67	119.97	118.47	189.10	186.40	189.50	73.10	72.05	73.25
9.24	130.43	131.69	131.38	119.56	120.63	119.83	173.70	172.20	174.50	72.96	72.33	73.24
13.92	131.80	131.18	131.39	121.02	121.57	120.06	153.10	153.10	153.90	71.39	71.39	71.77

**Figure 7:**
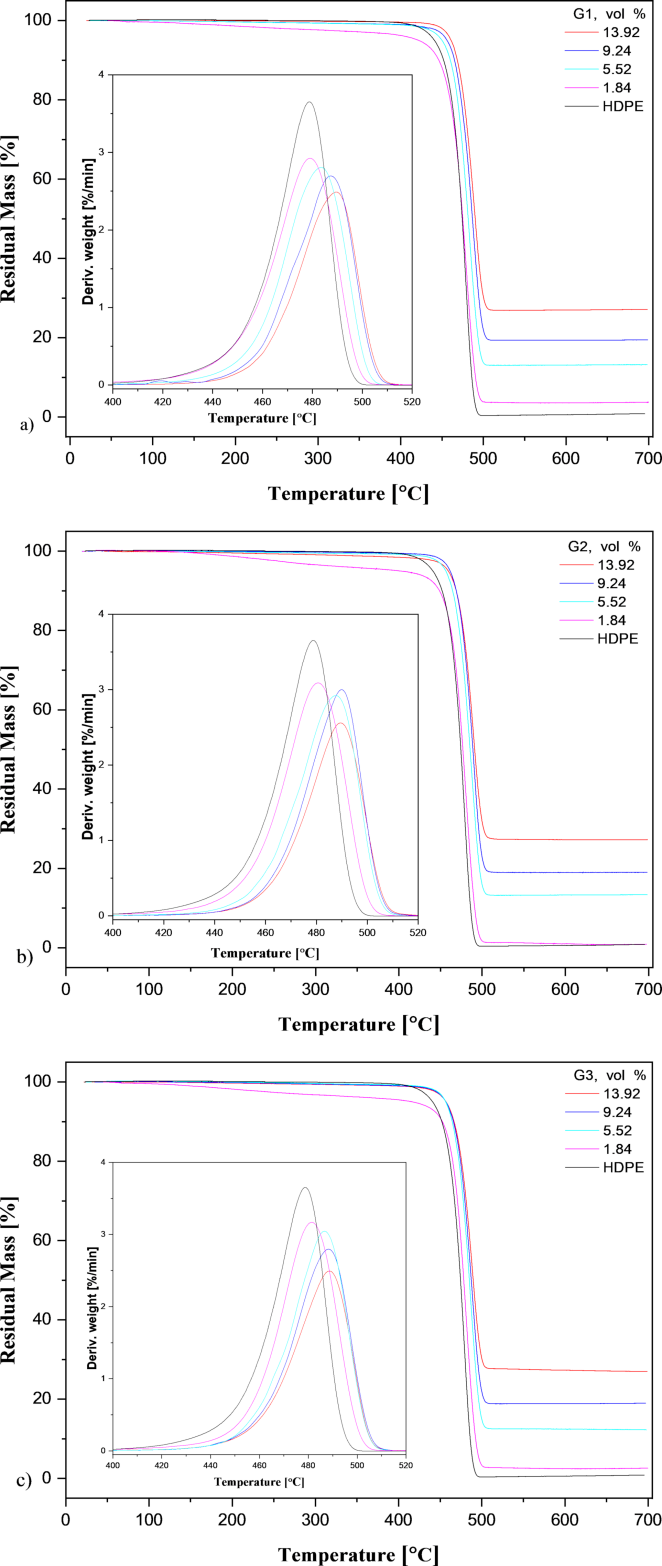
TGA thermographs of the HDPE/GnP nanocomposites with a) G1, b) G2, and c) G3 filler. The insets show derivative thermogravimetric (DTG) curves.

The parameters for the thermal stabilities predicted by TGA are given in Table 3. TGA curves of the samples are shown in Figure 7. Figure 7 shows that pure HDPE and the three HDPE/GnP nanocomposites all demonstrated a single-step thermal decomposition behavior between almost 440 °C and 500 °C, indicating that the increment of the GnPs could not affect the thermal decomposition behavior of the pure matrix. It was clear that the HDPE/GnP nanocomposites demonstrated better thermal stability than pure HDPE. The thermal stability of the HDPE-based nanocomposites decreased at a low volume fraction of GnP. This may have been due to agglomeration of the GnPs in the polymer matrix [8]. The agglomeration of the GnPs may have increased the molecular mobility and decreased the thermal stability of the polymer nanocomposites [39]. The thermal stability of the nanocomposites was enhanced by increasing the content of all three GnPs. This was attributed to the high thermal stability of the GnPs, the GnPs’ shielding effect on the combustion gas diffusion into and out of the polymer during its thermal decomposition [18], the barrier effect of the GnPs, the removal of free radicals, which started polymer decomposition, and uniform dispersion of the GnPs [8].

**Table 3:** Results of the TGA analysis of the HDPE/GnP nanocomposites.

GNP (vol %)	*T*_5%_ (°C)			*T*_50%_ (°C)			*T*_deg_ (°C)			residual mass (%)		
	HDPE/G1	HDPE/G2	HDPE/G3	HDPE/G1	HDPE/G2	HDPE/G3	HDPE/G1	HDPE/G2	HDPE/G3	HDPE/G1	HDPE/G2	HDPE/G3

0	440.77	440.77	440.77	474.44	474.44	474.44	478.79	478.79	478.79	0.84	0.84	0.84
1.84	421.66	425.42	415.48	475.11	476.86	478.31	479.07	480.62	481.45	3.88	1.53	3.09
5.52	449.97	456.34	456.46	481.68	485.53	485.06	483.55	487.62	486.63	13.19	13.52	11.68
9.24	456.05	461.50	456.16	486.82	488.91	487.37	487.31	489.92	488.27	19.58	19.02	19.24
13.92	461.05	459.45	457.16	490.19	490.99	489.64	489.52	489.54	488.64	27.44	27.31	26.75

Another reason was the hindering influence of the GnP layers on the diffusion of small gaseous molecules across the HDPE-based nanocomposites [40]. Generally, *T*_5%_ (5% sample weight loss) was defined as the onset degradation temperature [17]. The *T*_5%_ value of pure HDPE was 440.77 °C, while this value increased to 461.05 °C, 459.45 °C, and 457.16 °C, respectively, for the HDPE/G1, HDPE/G2, and HDPE/G3 nanocomposites. This showed that the GnPs had a positive influence on the thermal stability of HDPE-based nanocomposites [40]. For three types of HDPE/GnP nanocomposites, *T*_deg_ (maximum weight loss) increased by approximately 11 °C compared to the pure HDPE matrix (478.79 °C). The addition of GnPs increased this temperature; however, the size and concentration of the GnPs did not greatly influence *T*_deg_. The residual mass of the HDPE-based nanocomposites increased with increasing GnP loading, which was attributed to the existence of a carbon net structure in the HDPE/GnP nanocomposites [40] because the initial thermal degradation temperature of the GnPs was above 600 °C [15]. However, the GnP size did not have a significant effect on *T*_50%_, *T*_deg_, and the residual mass of the HDPE-based nanocomposites with GnPs, rather, it only affected *T*_5%_.

## Conclusion

The HDPE-based nanocomposites with three types of GnPs (G1, G2, and G3), having different thicknesses and lateral sizes, were prepared by a melt mixing method and compression molding. The size effects of the GnPs on the morphological, thermal, electrical, and mechanical properties of the composites was studied. The small differences in the GnPs’ surfaces’ chemical composition were detected by XPS, and the highest amount of oxygen that was found was 2.6 atom % for G1. FTIR and XRD findings showed that the addition and size of GnPs led to slight differences in the FTIR spectra of the nanocomposites compared to that of pure HDPE, while the addition of GnPs affected the reflection peaks and peak intensities. Based on SEM images, all GnPs showed uniform and random dispersion in the HDPE matrix. In particular, G2 displayed more GnP pathways, while G3 displayed a worse distribution than the other two. The lateral size of the GnPs was a more critical parameter for thermal and electrical conductivity enhancements of the nanocomposites than their thickness. Among all HDPE/GnP nanocomposites, the thermal and electrical conductivities of the HDPE/G2 nanocomposite were the highest, with 0.729 W/mK and 4.46 × 10^−6^ S/cm, corresponding to ≈89% and ≈11 orders of magnitude enhancement, respectively, compared to pure HDPE. As examined in terms of thickness, for a fixed lateral size, an optimal thickness was required to achieve the highest electrical and thermal conductivities. Additionally, the percolation threshold varied significantly with the size of the GnPs. G2 showed the lowest percolation threshold, and the nanocomposites with thinner GnPs exhibited lower percolation and higher electrical conductivity compared to those with thicker GnPs. Additionally, it was found that G1, with smaller lateral size and larger thickness (and the lowest aspect ratio and surface area), showed the lowest enhancement of the Young’s modulus and tensile strength due to worse distribution in the HDPE matrix and a less effective stress transfer between HDPE and GnPs. While a slight size effect of GnPs on *T*_m_, *T*_c_, Δ*H*_f_, and *X*_c_ of the nanocomposites was observed, the addition of GnPs influenced Δ*H*_f_ and *T*_c_; however, it did not affect *T*_m_ and *X*_c_. In addition, the thermal stability of the nanocomposites increased with increasing GnP content; however, the size of the GnPs only influenced *T*_5%_.

In summary, considering all the experimental measurements, G2 (having larger lateral size) seemed to be the most favorable type of GnP to achieve the best enhancement of nanocomposite properties.

## Experimental

### Materials

HDPE (LITEN FB 75, Unipetrol) with a density of 948 kg/m^3^ and a melt flow rate of 0.45 g/10 min (190 °C, 5 kg) was used as matrix material. Three different sizes of GnPs with trademark iGP (diameter (*D*) ≈ 5 μm, thickness (*t*) = 50–100 nm, aspect ratio (AR) = 50–100, surface area (SA) = 13 m^2^/g), iGP (*D* ≈ 44 μm, *t* = 50–100 nm, AR = 440–880, SA = 40 m^2^/g), and iGP2 (*D* ≈ 5 μm, *t* = 5–8 nm, AR = 625–1000, SA = 120–150 m^2^/g) were provided by Grafen Chemical Industries Co. and used as filler materials to investigate the size effect of GnPs on the properties of the HDPE/GnP nanocomposites. The GnPs with trademark iGP (*D* ≈ 5 μm), iGP (*D* ≈ 44 μm), and iGP2 (*D* ≈ 5 μm) were labelled G1, G2, and G3. All GNPs were used as supplied by the producer without any purification or functionalization process. The physical properties of the GNPs are shown in Table 4.

**Table 4:** Sizes of GnPs used in this study.

GnPs	lateral size (*D*, μm)	*t* (nm)	AR	SA (m^2^/g)

G1	≈5	50–100	50–100	13
G2	≈44	50–100	440–880	44
G3	≈5	5–8	635–1000	120–150

### Preparation of polymer nanocomposites

HDPE/GnP nanocomposites were produced by a melt mixing method using various amounts of filler up to 13.92 vol % (26.81 wt %) of GnP content. The volume fractions were calculated using the weight fraction of GnPs and densities of the pure constituents [1,3]. The HDPE-based nanocomposites were prepared in a Brabedender Plasticorder PLE 331 apparatus with a 30 mL mixing chamber at 160 °C for 15 min at 35 rpm. The samples for measurements were prepared by compression molding using a hot press (Fontijne 200, Fontijne Presses) at 180 °C without pressure for 2 min and under pressure (40 kPA) for 3 min, then solidified by air cooling.

### Characterization

X-ray photoelectron spectroscopy (K-alpha X-ray photoelectron spectrometer (XPS) system, Thermo Fisher Scientific) analysis was used to characterize the surface properties of the GnPs. The GnP and nanocomposite morphologies were investigated by SEM (XL 30S FEG electron microscope, Philips). The nanocomposite microstructures were examined at a cryofractured surface in liquid nitrogen, and then coated with a thin layer of gold. XRD analyses (X’Pert PRO, Philips) were performed to determine the overall phase and crystalline structure of the samples. The XRD patterns were recorded at 5–85°, with a scanning speed of 0.03°/s. FTIR measurements were carried out with a Thermo Fisher Scientific iS10 infrared spectrometer in the range of 4000–650 cm^−1^ at room temperature.

Broadband dielectric spectroscopy (BDS) measurements were conducted using a Novocontrol Concept 40 instrument with an Alpha dielectric spectrometer supplied by Novocontrol Technologies GmbH. A BDS-1200 parallel-plate capacitor with two gold-plated electrodes was used as a test cell for the samples and provided by Novocontrol Technologies. The diameter and thickness of the samples was 20 mm and 0.5 mm, respectively. All measurements were carried out in the 0.1 Hz–1 MHz frequency range at room temperature.

The thermal diffusivity of the samples was measured by the PTR technique, which is a nondestructive characterization method based on the detection of an IR signal, called the photothermal signal, emitted after a change of the sample’s temperature [41]. The heating is achieved by a frequency-modulated laser beam (DPSS, Dream Lasers Technol. Co., model SDL-532-300T) with 300 mW at 532 nm wavelength and a diameter of 0.6 mm at 1/e. The IR radiations linked to the fast conversion of a fraction of the absorbed optical energy into heat were collected by a suitable optic (Au-coated mirrors, Edmund Optics) and focused on an IR photodetector (HgCdTe photoconductive detector, Kolmar Technologies, KMPV 11-1-J1/DC). Afterwards, a lock-in amplifier (Stanford Research Systems SR865) output the amplitude and phase of the detector’s electrical signal proportional to the variation of the sample surface temperature, which depended on its thermophysical properties. A more detailed description of the setup used can be found elsewhere [42]. Using this PTR setup, different types of material (nanocomposites [3], organic [43–45], irradiated [46], metal–semiconductor couples [42,47], etc.) can be thermally characterized to determine thermophysical properties, such as the thermal diffusivity and thermal effusivity. At lower frequencies, the measurements are subject to 3D effects and necessitate a change in the laser beam shape. At low frequencies, a flat top (FT) optics grouping consisting of a diffuser/beam-shaper (ED1-C20, diameter 1*"*, 20°, Circle Pattern Diffuser, Thorlabs) and two lenses (one to collect the large diffused beam and another to converge it on the sample) was used. Using this configuration, the shape of the heating spot was circular, and the illumination was uniform on the sample’s surface. The heating was therefore considered 1D, and a 1D thermal characterization of the HDPE samples could be applied by analyzing the amplitude and phase of the PTR signal using a 1D quadrupole-based model [48–49]. This modeling was used by a Gauss–Newton algorithm to simultaneously adjust the experimental amplitude and phase of the PTR signal analyzed by the lock-in amplifier to extract the thermal diffusivity and optical absorption coefficient of the studied samples.

The tensile tests were conducted using an Instron 4301 tensile test machine at a crosshead speed of 10 mm/min at room temperature, according to ASTM D638. The samples were cut in dog-bone shapes with a thickness of 1 mm, a width of 4 mm, and a gauge length of 30 mm by a pneumatic press (APK 4L, Tinius Olsen LTD). The mean values of seven tests were reported for each sample.

Thermogravimetric analysis (TGA) of the samples was performed using a Q500 system from TA Company. TGA was carried out at a heating rate of 10 °C/min from room temperature to 700 °C under a nitrogen atmosphere.

Differential scanning calorimetry (DSC) analysis was performed using a modulated differential scanning calorimeter (Q200 series MDSC, TA instruments) under a nitrogen atmosphere in the −10 °C–200 °C range. Two heating cycles were performed for each sample. The samples were initially heated from −10 °C–200 °C at 10 °C/min^−1^ and held at 200 °C for 1 min to eliminate the thermal history of the samples. Then, they were cooled to −10 °C at the same rate and held for 1 min at this temperature. Finally, the samples were reheated to 200 °C in the MDSC mode, with a temperature oscillation of 1.326 °C amplitude, a modulating period (*P*) of 100 s, and a heating rate of 5 °C/min. The degree of crystallinity (*X*_c_) of the samples was determined through Equation 1:

[1] $$X_{c}=\;\Delta H\times \;100/\left(\Delta H_{0}\times \;\left(1\;-\;\varphi \right)\right)$$

where Δ*H* was the heat of fusion of the sample, Δ*H*_0_ = 293 J/g was the heat of fusion for 100% crystalline HDPE [50], and φ referred to the weight fraction of the filler in each sample.

## Supporting Information

File 1 FTIR spectroscopy and XRD patterns of HDPE/GnP nanocomposites with various concentrations of GnPs.
