# Supplementary material for: Size effects of graphene nanoplatelets on the properties of high-density polyethylene nanocomposites: morphological, thermal, electrical, and mechanical characterization
Source: Beilstein J Nanotechnol. 2020 Jan 14;11:167–79. doi: 10.3762/bjnano.11.14 (PMC7006480; doi:10.3762/bjnano.11.14)
Supplement: File 1 — FTIR spectroscopy and XRD patterns of HDPE/GnP nanocomposites with various concentrations of GnPs. [file Beilstein_J_Nanotechnol-11-167-s001.pdf]

BEILSTEIN JOURNAL OF NANOTECHNOLOGY

Supporting Information

for

Size effects of graphene nanoplatelets on the properties of high-density polyethylene nanocomposites: morphological, thermal, electrical, and mechanical characterization

Tuba Evgin, Alpaslan Turgut, Georges Hamaoui, Zdenko Spitalsky, Nicolas Horny, Matej Micusik, Mihai Chirtoc, Mehmet Sarikanat and Maria Omastova

Beilstein J. Nanotechnol. **2020,** *11,* 167–179. doi:10.3762/bjnano.11.14

FTIR spectroscopy and XRD patterns of HDPE/GnP nanocomposites with various concentrations of GnPs

License and Terms: This is a supporting information file under the terms of the Creative Commons Attribution License (https://creativecommons.org/licenses/by/4.0). Please note that the reuse, redistribution and reproduction in particular requires that the authors and source are credited.
The license is subject to the *Beilstein Journal of Nanotechnology* terms and conditions: (https://www.beilstein-journals.org/bjnano)

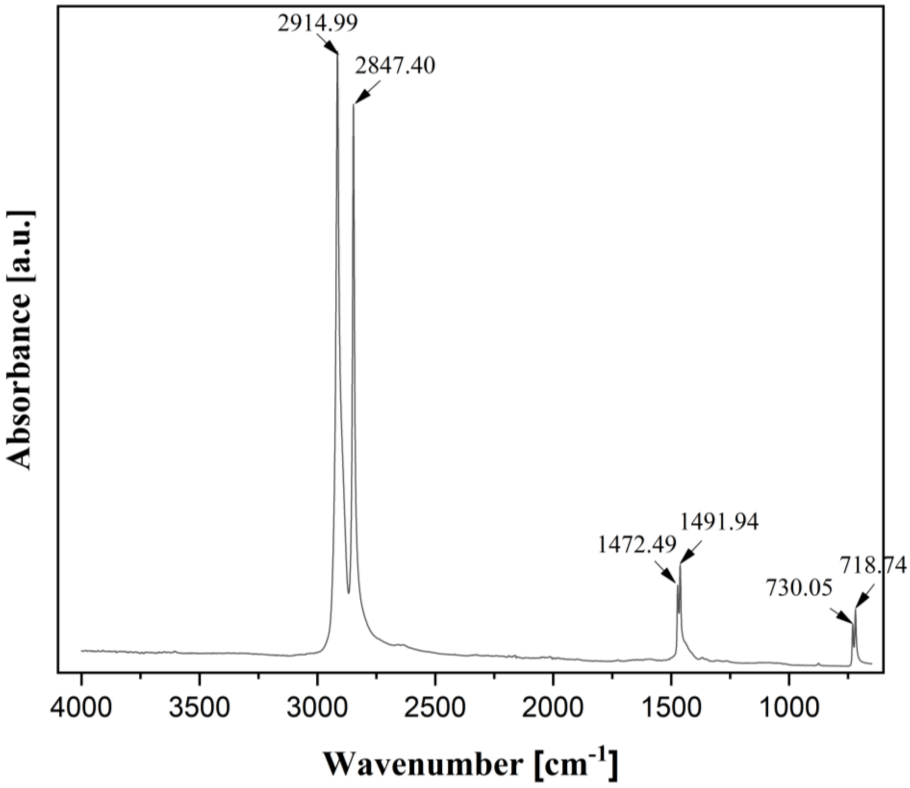

Figure S1: FTIR spectroscopy of HDPE.

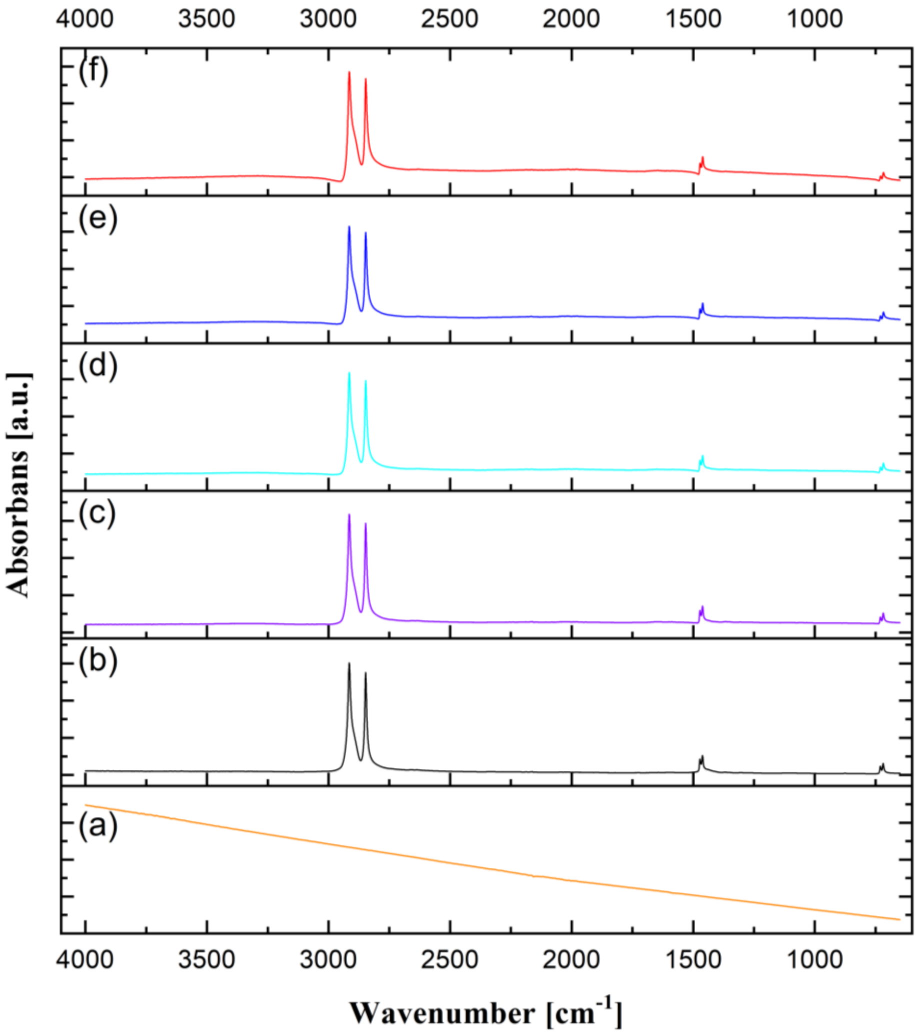

Figure S2: FTIR spectroscopy of a) G1, b) HDPE, c) HDPE/1.84 vol % G1, d) HDPE/5.52 vol % G1, e) HDPE/9.24 vol % G1, and f) HDPE/13.92 vol % G1 nanocomposite.

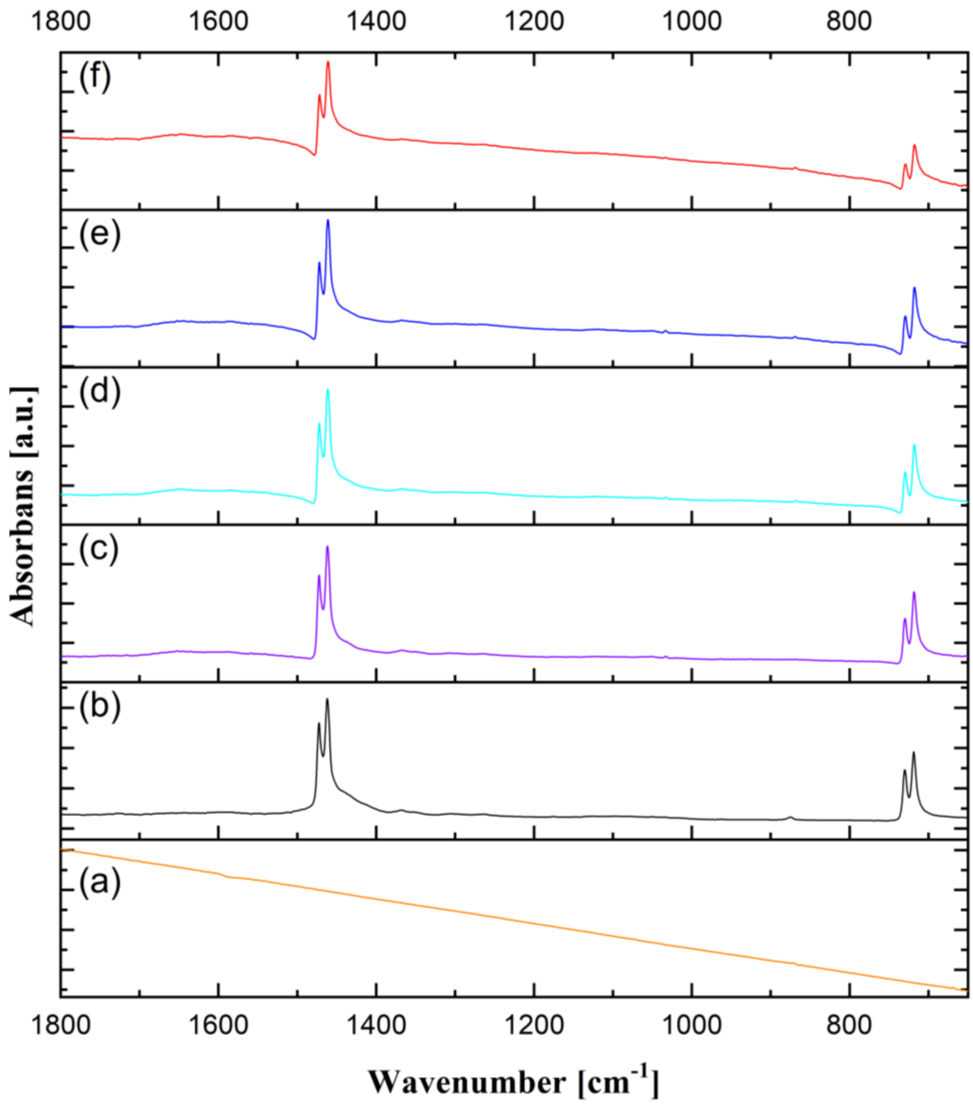

Figure S3: FTIR spectroscopy of a) G1, b) HDPE, c) HDPE/1.84 vol % G1, d) HDPE/5.52 vol % G1, e) HDPE/9.24 vol % G1, and f) HDPE/13.92 vol % G1 nanocomposite in the range of 650–1800 cm^{-1}.

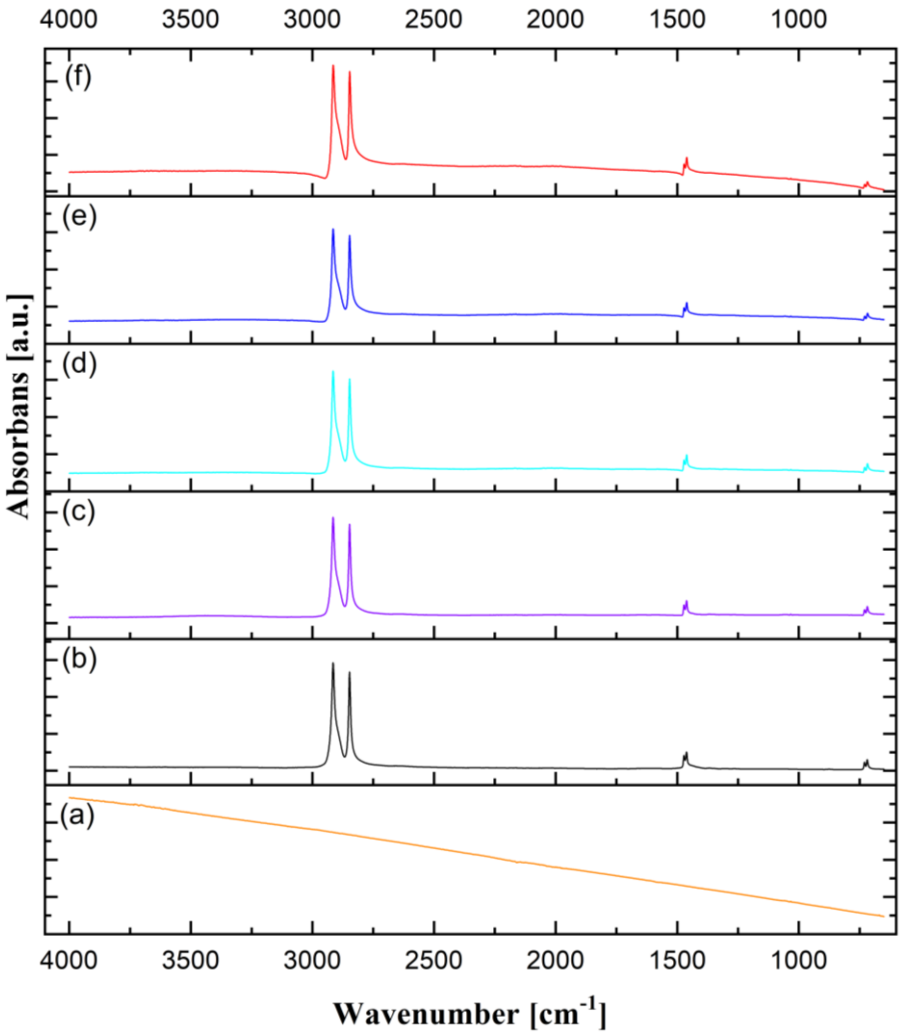

Figure S4: FTIR spectroscopy of a) G2, b) HDPE, c) HDPE/1.84 vol % G2, d) HDPE/5.52 vol % G2, e) HDPE/9.24 vol % G2, and f) HDPE/13.92 vol % G2 nanocomposite.

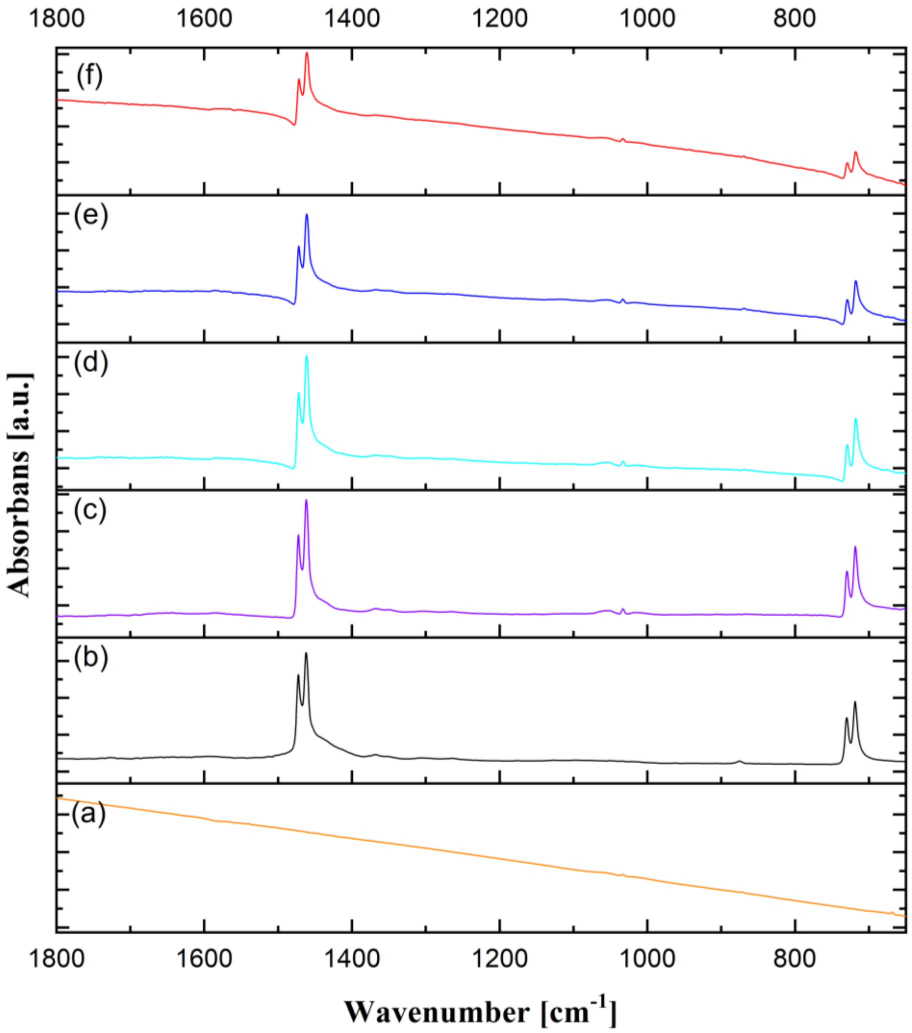

Figure S5: FTIR spectroscopy of a) G2, b) HDPE, c) HDPE/1.84 vol % G2, d) HDPE/5.52 vol % G2, e) HDPE/9.24 vol % G2, and f) HDPE/13.92 vol % G2 nanocomposite in the range of 650–1800 cm^{-1}.

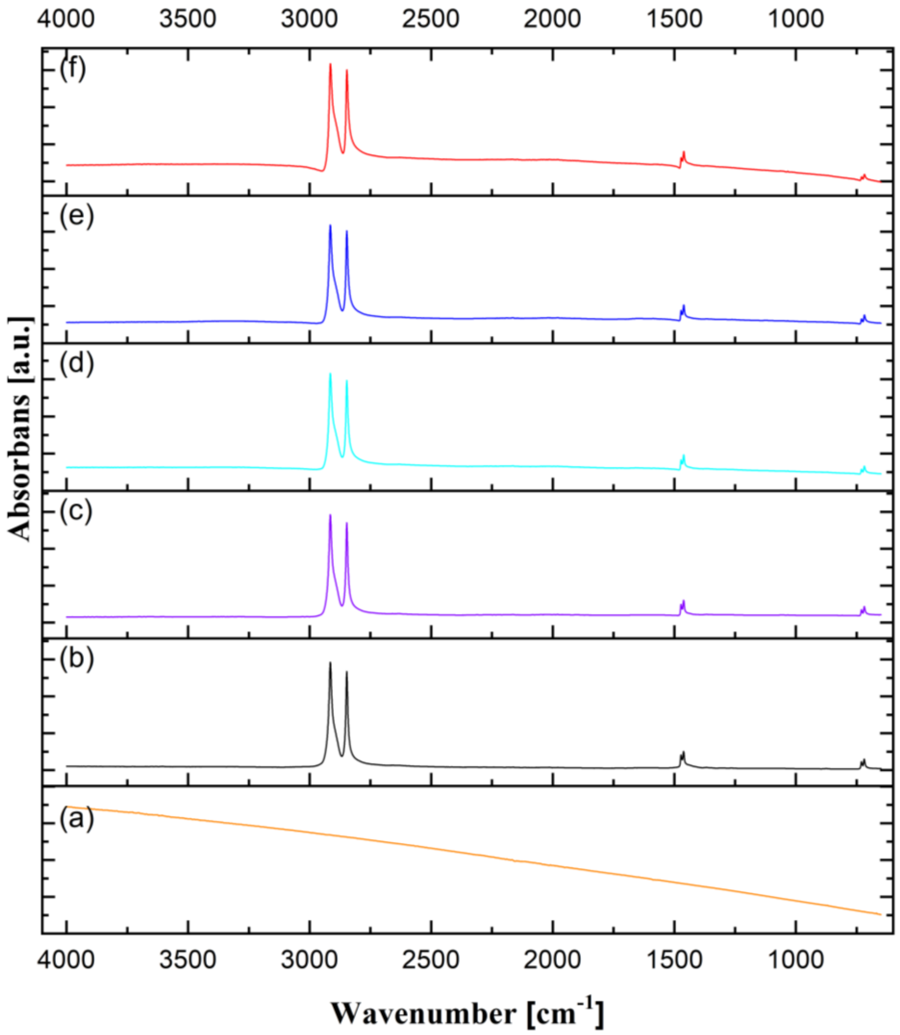

Figure S6: FTIR spectroscopy of a) G3, b) HDPE, c) HDPE/1.84 vol % G3, d) HDPE/5.52 vol % G3, e) HDPE/9.24 vol % G3, and f) HDPE/13.92 vol % G3 nanocomposite.

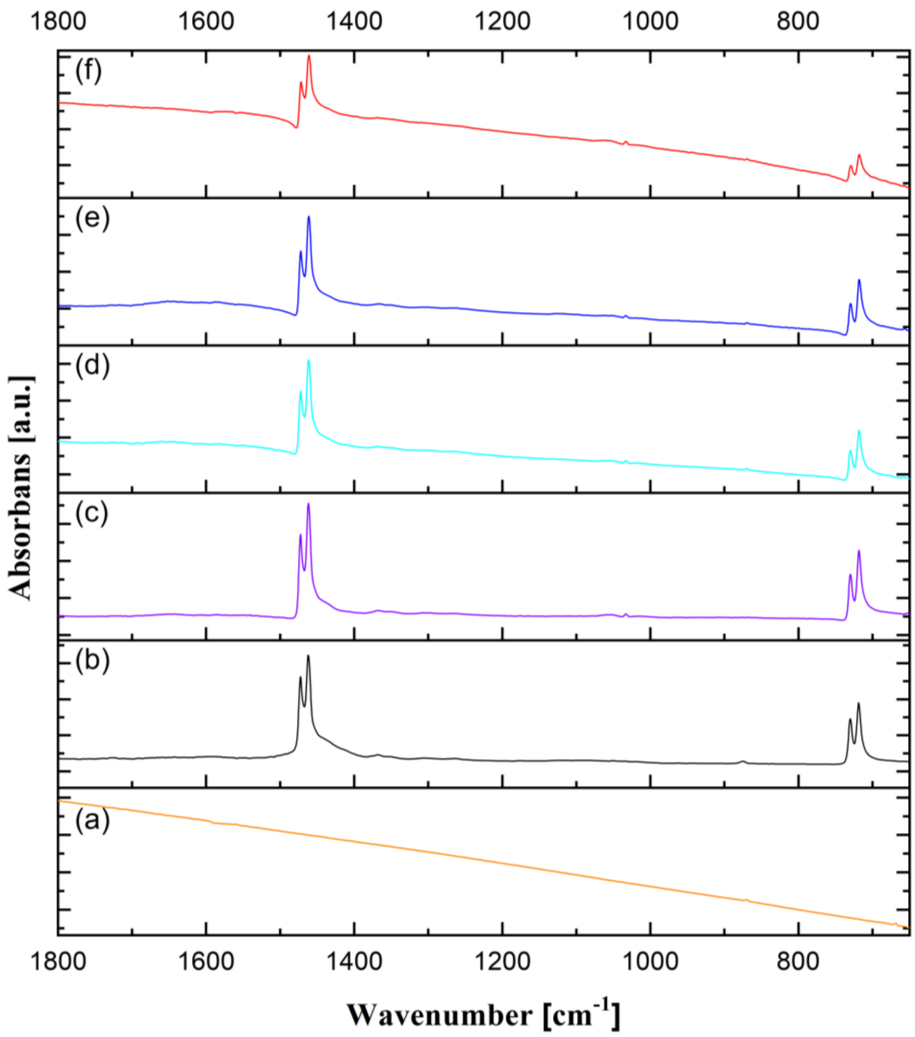

Figure S7: FTIR spectroscopy of a) G3, b) HDPE, c) HDPE/1.84 vol % G3, d) HDPE/5.52 vol % G3, e) HDPE/9.24 vol % G3, and f) HDPE/13.92 vol % G3 nanocomposite in the range of 650–1800 cm^{-1}.

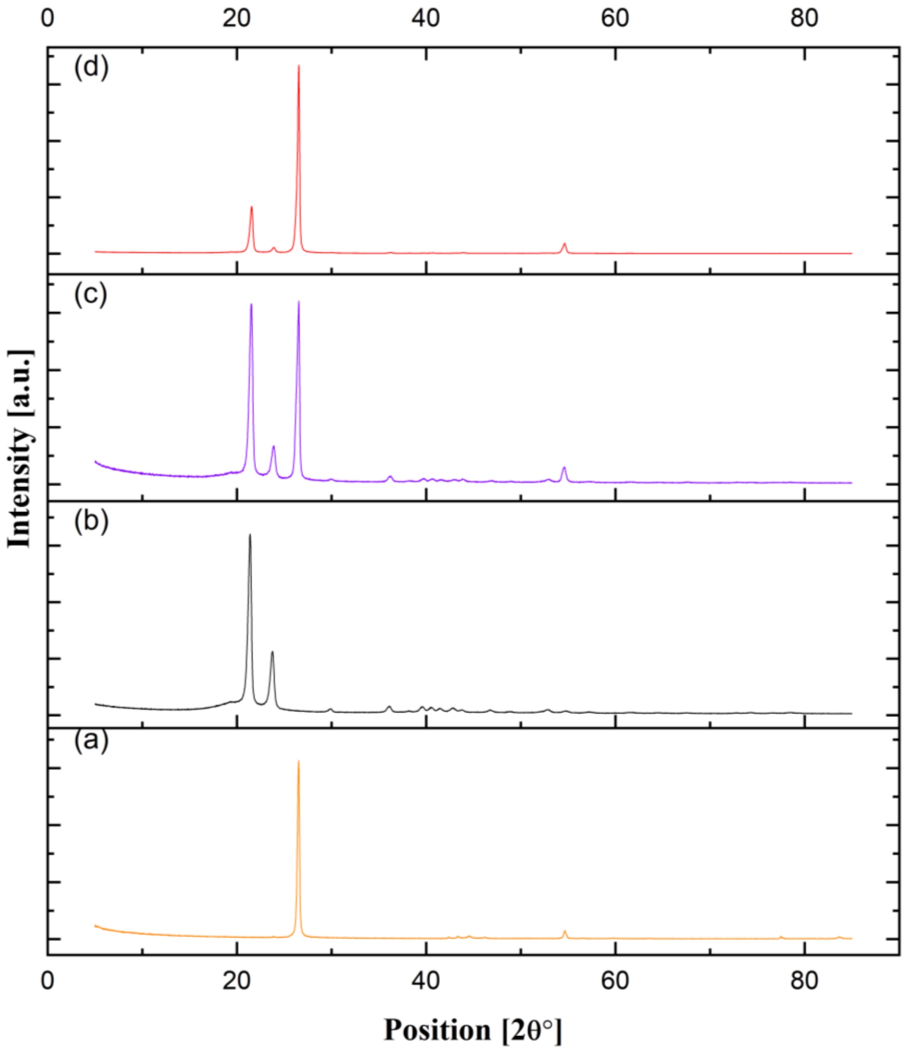

Figure S8: XRD pattern of a) G1, b) HDPE, c) HDPE/1.84 vol % G1, d) HDPE/13.92 vol % G1 nanocomposite.

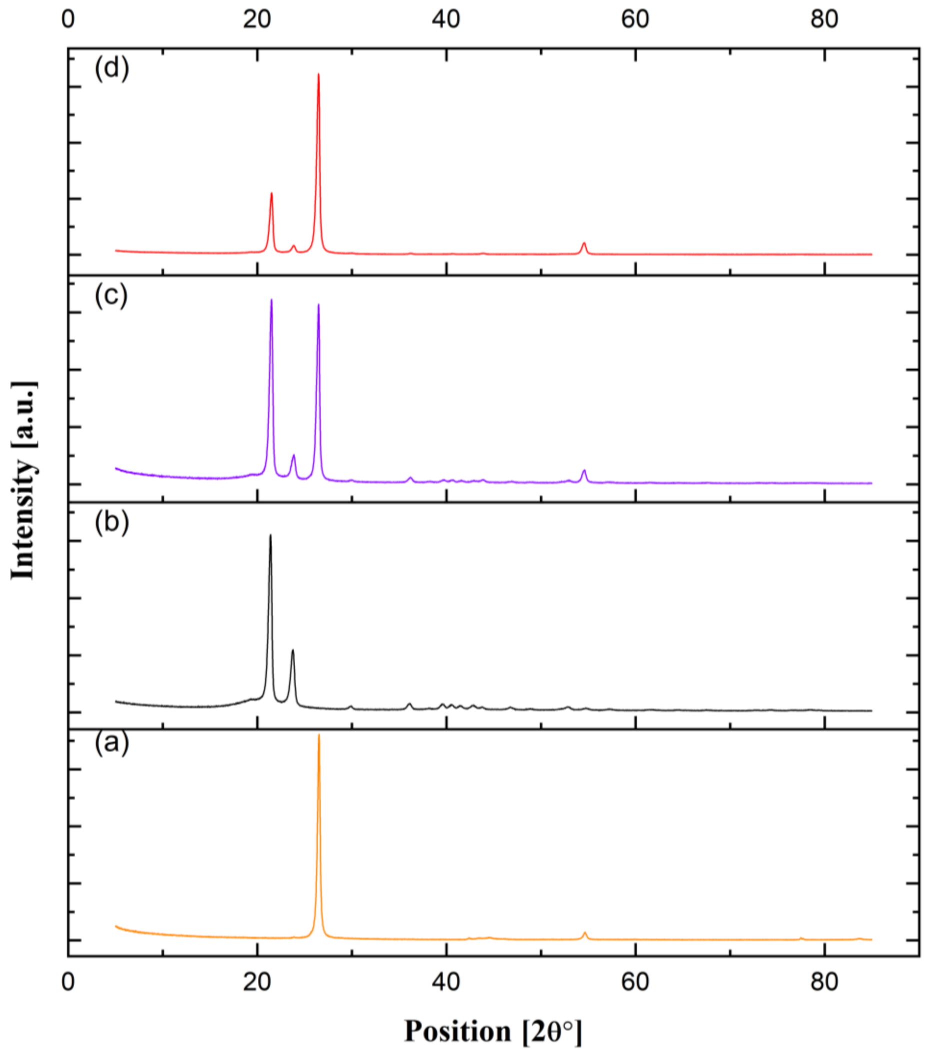

Figure S9: XRD pattern of a) G2, b) HDPE, c) HDPE/1.84 vol % G2, d) HDPE/13.92 vol % G2 nanocomposite.

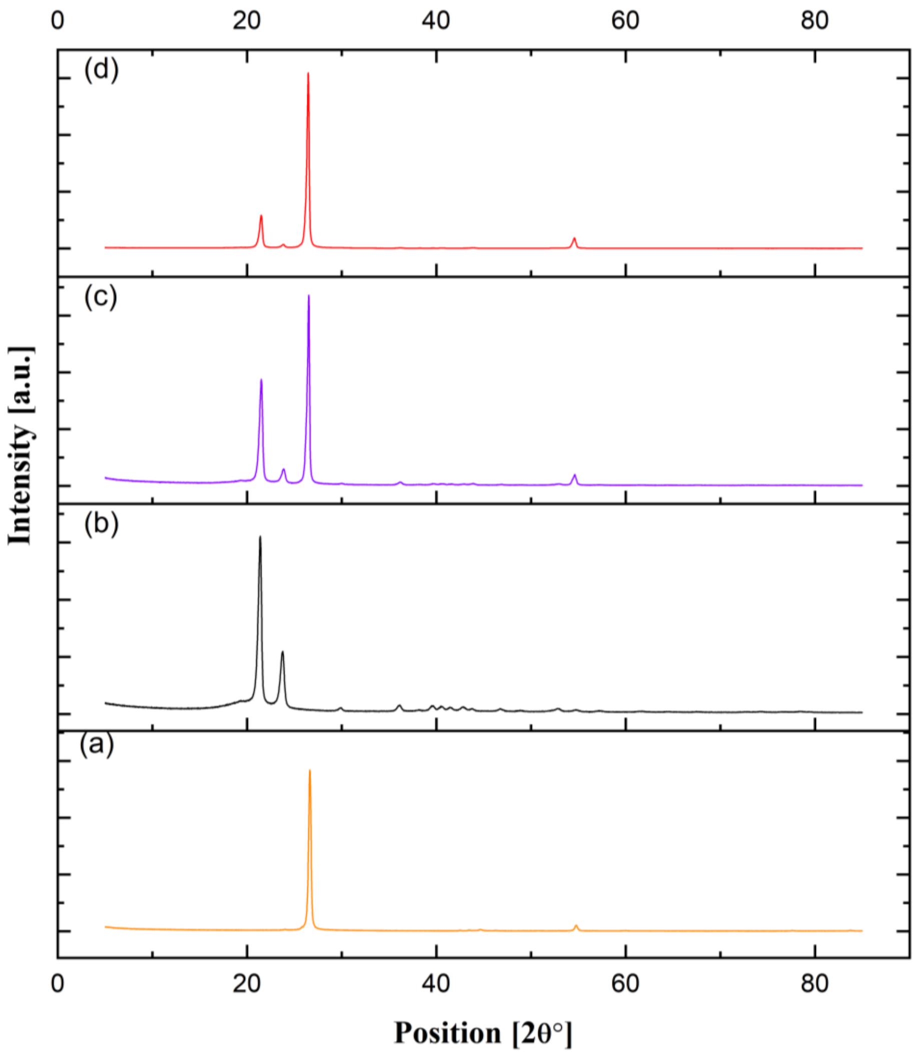

Figure S10: XRD pattern of a) G3, b) HDPE, c) HDPE/1.84 vol % G3, d) HDPE/13.92 vol % G3 nanocomposite.
